# Nanotechnology in Cutaneous Oncology: The Role of Liposomes in Targeted Melanoma Therapy

**DOI:** 10.3390/molecules31020344

**Published:** 2026-01-19

**Authors:** Ellen Paim de Abreu Paulo, Laertty Garcia de Sousa Cabral, Jean-Luc Poyet, Durvanei Augusto Maria

**Affiliations:** 1 Faculty of Medicine, University of Sao Paulo (FMUSP), Sao Paulo 05403-000, Brazil; ellen.paulo.esib@esib.butantan.gov.br (E.P.d.A.P.); laertty.c@alumni.usp.br (L.G.d.S.C.); 2 Laboratory of Development and Innovation, Butantan Institute, Sao Paulo 05585-000, Brazil; 3 INSERM UMRS1342—CNRS EMR8000, Institut de Recherche Saint-Louis, Hôpital Saint-Louis, 75010 Paris, France; 4 Université Paris Cité, 75006 Paris, France

**Keywords:** melanoma, targeted therapy, nanotechnology, liposomal platforms

## Abstract

Melanoma is an aggressive skin cancer that continues to present major therapeutic difficulties. Although targeted drugs and immune checkpoint inhibitors have improved outcomes, resistance and treatment-related toxicity limit long-term benefit. In recent years, nanotechnology has been explored as a way to improve how drugs are delivered and to achieve greater tumor selectivity. Among available nanocarriers, liposomes have attracted particular interest. Built from lipid bilayers, they can carry both hydrophilic and hydrophobic molecules, and they are generally well tolerated. Importantly, their surface can be modified with polymers or targeting ligands to direct the carrier more selectively to melanoma cells. Experimental models show that liposomal drug formulations can increase concentrations in tumor tissue while limiting distribution to healthy organs. They have also been used successfully to combine different types of agents, chemotherapies, immunomodulators, and nucleic acids, within a single delivery system. These findings suggest genuine potential to address several of the shortcomings of conventional treatments. Although translation to the clinic is slowed by challenges such as formulation stability and large-scale production, liposomes represent an important step toward safer and more effective melanoma therapy within the broader field of oncologic nanotechnology.

## 1. Introduction

Cutaneous melanoma is one of the most aggressive and lethal forms of skin cancer, due to its high invasiveness and resistance to multiple lines of therapy [1,2]. Although it accounts for only about 5% of all skin cancer cases, melanoma is responsible for over 75% of skin cancer-related deaths, particularly in advanced stages [3,4,5]. Over the past decade, the introduction of immuno-oncological approaches and targeted therapies has profoundly changed the treatment landscape. Nonetheless, these therapies are often imperfect in terms of clinical responses or long-term outcomes [6,7,8].

Several factors contribute to the therapeutic refractoriness observed in melanoma. Recurrent genetic mutations (notably in BRAF, NRAS, and KIT) [9,10,11,12], activate oncogenic signaling cascades including the MAPK and PI3K/AKT pathways, while up-regulation of anti-apoptotic proteins and immune evasion further diminish therapeutic efficacy [13,14,15]. Furthermore, the heterogeneous nature of the tumor microenvironment such as variations in pH, oxygen levels, irregular vascularization, and dense extracellular matrix limits drug penetration and facilitates the persistence of resistant cellular niches [9,13,15].

Nanotechnology is increasingly viewed as a promising avenue in cutaneous oncology, offering strategies to improve precision, safety, and therapeutic effectiveness in melanoma treatment [16].

Among the available nanocarriers, liposomes have drawn particular attention for their capacity to modify pharmacokinetics and biodistribution, protect drugs from enzymatic degradation, and encapsulate both hydrophilic and lipophilic agents [17,18]. Their bilayer lipid-based biocompatible structure and functional versatility are particularly well suited for oncological applications, with several formulations already approved for clinical use in other cancer types [18]. Liposomes have been explored in melanoma therapy as versatile carriers for the targeted delivery of established cytotoxic drugs, such as doxorubicin and paclitaxel, as well as kinase inhibitors, immunomodulators, and peptide- or RNA-based vaccines [19,20,21]. Surface functionalization of liposomes with monoclonal antibodies, peptides or specific ligands, resulting in immunoliposomes, allows for active targeting of tumor-specific or immune-related receptors, thereby enhancing therapeutic efficacy and reducing adverse effects [22]. Moreover, the development of liposomes responsive to internal stimuli (e.g., acidic pH, glutathione) or external triggers (e.g., temperature, light) has enabled the creation of controlled and stimulus-responsive drug-delivery systems, optimizing both the timing and localization of drug release [23,24]. These strategies are particularly beneficial in tumors like melanoma, which display a highly dynamic and complex tumor microenvironment [13].

Despite encouraging progress, translation to clinical practice remains difficult. Barriers include limited dermal penetration, intra and intertumoral variability, manufacturing complexity, and regulatory requirements [25,26]. Nonetheless, ongoing progress in formulation, characterization, and liposomal surface engineering combined with the integration of personalized medicine tools such as pharmacogenomics and bioinformatics are likely to support liposomal technology integration in melanoma management [26].

In this review, we will examine the latest developments regarding the use of liposomes as targeted drug delivery systems for melanoma therapy. We will highlight their technological foundations, therapeutic applications, up to date experimental and clinical outcomes as well as the main challenges and future perspectives in the field of cutaneous oncology.

## 2. Epidemiology and Classification of Melanoma

Melanocytes, pigment-producing cells of neural crest origin predominantly located in the basal epidermal layer, constitute the cellular source of cutaneous melanoma [27,28]. Over the last decades, the global incidence of melanoma has steadily increased, particularly in countries with fair-skinned populations exposed to intermittent and intense ultraviolet (UV) radiation, such as Australia, New Zealand, North America, and parts of Northern Europe [4,29]. Recent data from GLOBOCAN and the American Cancer Society confirm that this malignancy continues to significantly affect adults over the age of 50, although it also presents a high prevalence among younger adults, particularly women between 20 and 39 years of age [30].

From a clinical and histopathological standpoint, melanoma is classified into subtypes based on growth patterns, anatomical distribution, and specific morphological characteristics [31,32]. The most common subtype is superficial spreading melanoma, accounting for approximately 60% to 70% of cases [33]. It is characterized by a prolonged radial growth phase before vertical invasion and typically appears as flat or slightly raised lesions with irregular pigmentation and poorly defined borders, predominantly on intermittently sun-exposed skin [34]. Nodular melanoma, the second most common subtype, is defined by rapid vertical growth without a discernible radial phase. It usually manifests as a well-demarcated pigmented nodule and is associated with a poorer prognosis due to early dermal invasion and aggressive behavior [35]. Lentigo maligna melanoma, more frequent in elderly patients, occurs in chronically sun-exposed areas such as the face and neck. It evolves from lentigo maligna (in situ melanoma) and exhibits a slow growth rate with a long radial phase before dermal invasion [36]. In contrast, acral lentiginous melanoma, although rare in Caucasian populations, is more prevalent among individuals with darker skin tones and typically arises on the palms, soles, and subungual regions, often being diagnosed at more advanced stages [37]. Other less common variants include desmoplastic melanoma [38], amelanotic melanoma [39], and mucosal melanomas [40], each requiring specific diagnostic and therapeutic approaches.

Clinical staging of melanoma follows the American Joint Committee on Cancer (AJCC) TNM classification, currently in its eighth edition, which considers tumor thickness (Breslow index), ulceration, mitotic rate, lymph node involvement, and distant metastasis [41,42,43]. Localized melanomas (stages I and II) generally have a favorable prognosis, with five-year survival rates exceeding 90% [44,45]. However, survival decreases substantially with disease progression: approximately 65% for regional disease (stage III) and less than 25% for metastatic melanoma (stage IV), even with recent advances in immunotherapies and targeted treatments [45]. Additional prognostic indicators include lymphovascular invasion, the presence of tumor-infiltrating lymphocytes, and specific molecular alterations such as mutations in the BRAF, NRAS, and c-KIT genes, which not only impact prognosis but also inform therapeutic decisions and the development of emerging drug delivery technologies [46,47,48].

Understanding melanoma classification and clinical determinants is essential not only for conventional oncological management but also for the formulation of innovative therapeutic strategies [48]. The complexity of histological subtypes and the diverse clinical behavior of melanoma justify investments in highly specific approaches, such as functionalized liposomes, which are being tested as targeted delivery systems for distinct tumor profiles and as potentially effective tools to address tumor heterogeneity and resistance to standard therapies [46,47].

## 3. Current Therapeutic Landscape of Melanoma: Limitations and the Search for Innovation

Although major progress has been made in understanding the molecular biology of melanoma, the treatment of this malignancy especially in its advanced stages remains difficult [49,50]. Localized tumors are typically managed through surgical excision, but systemic treatment relies on several additional modalities. These include immune checkpoint inhibitors (anti-CTLA-4 or anti-PD-1 agents), targeted therapies that act on driver mutations such as BRAF and MEK, and, in some cases, radiotherapy or classic chemotherapy [51,52,53].

**Figure 1:**
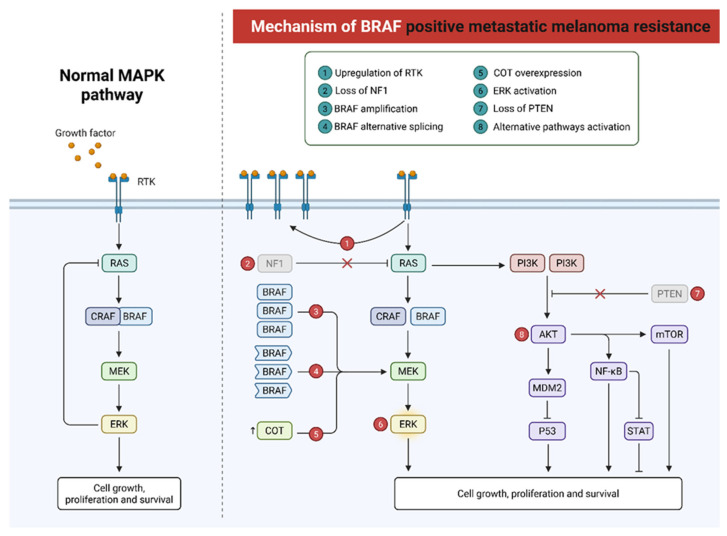
Contextual schematic representation of the normal MAPK pathway (**left**) and the mechanisms of resistance in BRAF-positive metastatic melanoma (**right**). On the left panel, physiological activation of the MAPK cascade occurs through receptor tyrosine kinases (RTKs), RAS, RAF, MEK, and ERK, leading to cell growth, proliferation, and survival. On the right panel, the main mechanisms of acquired resistance to BRAF inhibitors are shown: (1) RTK upregulation, (2) NF1 loss, (3) BRAF amplification, (4) BRAF alternative splicing, (5) COT overexpression, (6) ERK reactivation, (7) PTEN loss, and (8) activation of alternative signaling pathways, including PI3K/AKT/mTOR. These alterations restore proliferative signaling, reducing therapeutic efficacy.

The introduction of BRAF inhibitors like vemurafenib and dabrafenib, often used together with MEK inhibitors such as trametinib has significantly improved outcomes for patients carrying activating MAPK pathway mutations found in roughly 40–60% of cutaneous melanomas (Figure 1) [51,52,54]. Yet, secondary resistance is common, with disease recurrence often occurring within 6 to 12 months of treatment initiation. Similarly, although immunotherapy has produced durable responses in a subset of patients, nearly half fail to respond or develop severe immune-related side effects [52]. Therapeutic efficacy is further compromised by immune-escape mechanisms, low tumor immunogenicity, heterogeneous antigen expression, and an immunosuppressive tumor microenvironment [53].

Chemotherapy, while historically utilized, offers limited efficacy in metastatic melanoma and significant systemic toxicity. Combined strategies such as dual checkpoint blockade (anti-PD-1 plus anti-CTLA-4) or targeted therapy combined with immunomodulation are actively investigated but still face resistance, cumulative toxicity, or heterogeneous response profiles issues [55,56,57].

**Figure 2:**
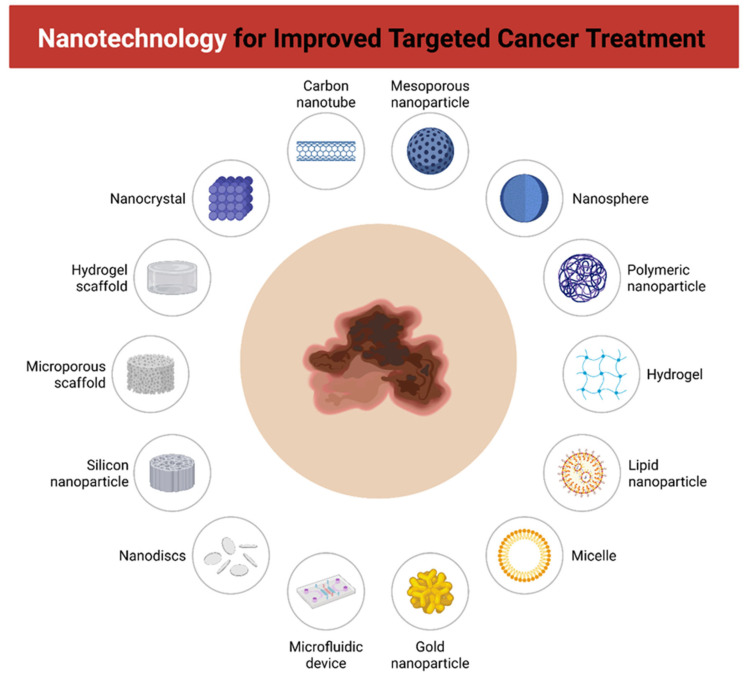
Contextual overview of nanotechnology-based platforms for improved targeted cancer therapy. Different nanostructures and devices are illustrated as potential carriers for drug delivery and tumor targeting, including carbon nanotubes, mesoporous nanoparticles, nanospheres, polymeric nanoparticles, hydrogels, lipid nanoparticles, micelles, gold nanoparticles, microfluidic devices, nanodiscs, silicon nanoparticles, microporous scaffolds, hydrogel scaffolds, and nanocrystals. These systems enhance therapeutic efficacy, stability, and specificity, while reducing systemic toxicity.

In this context, nanotechnology emerges as a promising alternative (Figure 2) [58,59,60,61]. Nanostructure’s ability to modulate drug pharmacokinetics, protect labile molecules from degradation, enable controlled release, and achieve active targeting of tumor cells or components of the tumor microenvironment position these platforms as potential drivers of a new generation of melanoma therapies [49,61,62].

## 4. Liposomal Formulations and Their Physicochemical Characteristics for Melanoma Therapy

**Figure 3:**
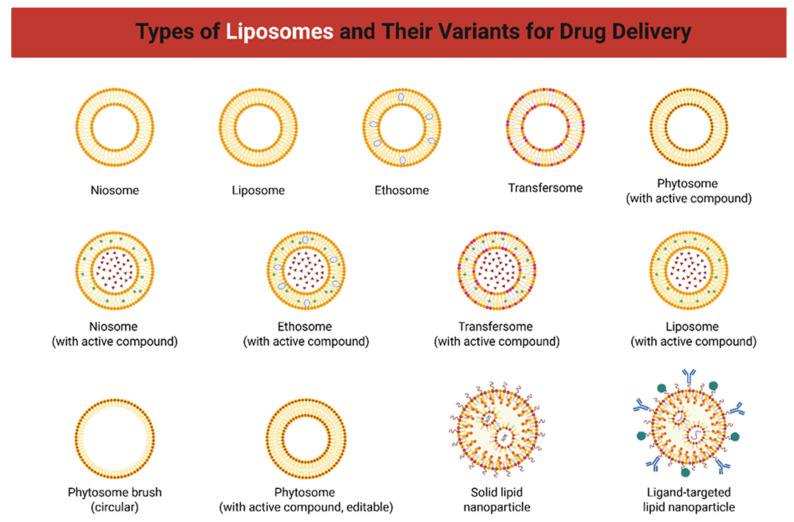
Types of liposomes and their variants used for drug delivery. Different lipid-based nanocarriers are represented, including niosomes, liposomes, ethosomes, transfersomes, phytosomes, circular phytosome brushes, solid lipid nanoparticles, and ligand-targeted lipid nanoparticles. Several formulations are illustrated with encapsulated active compounds, highlighting their role in enhancing drug stability, bioavailability, and targeted delivery.

Liposomal formulations developed for melanoma therapy are designed to optimize the pharmacokinetic and pharmacodynamic profiles of therapeutic agents while minimizing systemic toxicity and improving tumor specificity [63,64]. These nanosystems are based on spherical vesicles composed of one or more phospholipid bilayers surrounding an aqueous core, allowing the simultaneous encapsulation of hydrophilic compounds in the internal aqueous phase and lipophilic molecules within the lipid bilayer [65,66,67]. The composition of liposomes plays a central role in determining their biological performance, including drug loading efficiency, membrane stability, circulation time, and release kinetics (Figure 3) [68,69]. Commonly used lipids include phosphatidylcholine (PC), phosphatidylethanolamine (PE), phosphatidylglycerol (PG), and cholesterol. Cholesterol is incorporated into formulations to modulate bilayer fluidity and mechanical strength, reducing membrane permeability and increasing vesicle stability during systemic circulation [70,71].

Biocompatibility is a critical feature of liposomal systems, especially for oncological applications, as it ensures minimal immunogenicity and cytotoxicity toward non-target tissues [72,73,74]. Liposomes composed of naturally occurring or biologically inert phospholipids are generally well tolerated in vivo, and their structural resemblance to cellular membranes enhances cellular uptake via endocytosis [72]. Furthermore, surface modifications such as the covalent attachment of polyethylene glycol (PEG) chains to the liposomal surface are widely employed to confer a hydrophilic steric barrier, reducing opsonization and recognition by the mononuclear phagocyte system (MPS) [75,76,77,78]. This strategy significantly prolongs the systemic half-life of liposomes, enhancing their accumulation in tumor tissues through the enhanced permeability and retention (EPR) effect, a phenomenon particularly relevant in the context of melanoma due to its aberrant and leaky vasculature [77,78].

Although the EPR effect has been widely demonstrated in murine melanoma models, its relevance in human tumors is far more limited. Clinical imaging and pharmacokinetic studies indicate that the median fraction of injected nanoparticles reaching human tumors is typically below 1% of the administered dose, with marked interpatient and intratumoral heterogeneity. Recent work also suggests that active endothelial transcytosis may play a more significant role in nanoparticle entry than classical passive extravasation alone, underscoring the need for delivery strategies that do not rely exclusively on EPR when considering clinical translation in melanoma.

The physicochemical stability of liposomal formulations is influenced by multiple factors, including lipid composition, storage conditions, and the physicochemical nature of the encapsulated drug [68,79]. Instability may manifest as vesicle aggregation, leakage, hydrolysis of ester bonds in phospholipids, or oxidative degradation of unsaturated lipids [75]. The incorporation of antioxidants (e.g., α-tocopherol) and the use of saturated lipids with higher transition temperatures can improve stability under physiological conditions [79]. Additionally, lyophilization with cryoprotectants such as trehalose or sucrose is a commonly used strategy to enhance long-term storage stability without compromising vesicle integrity [80,81].

Encapsulation efficiency (EE) is a key parameter that reflects the capacity of liposomes to incorporate and retain the active pharmaceutical ingredient. EE is influenced by the hydrophilicity or lipophilicity of the drug, the method of liposome preparation (e.g., thin-film hydration, ethanol injection, microfluidics), lipid-to-drug ratio, and bilayer composition [82,83,84]. For hydrophilic agents, encapsulation occurs predominantly in the aqueous core, while lipophilic compounds are solubilized in the lipid bilayer. Optimization of these parameters is essential to achieve therapeutic drug concentrations at the tumor site and to prevent premature release during circulation [82,83].

Surface charge is an important parameter as it influences liposomes stability, cellular uptake, and biodistribution [85,86]. Neutral or slightly negative liposomes typically evade rapid clearance and exhibit longer circulation times. Positively charged liposomes enhance cellular uptake due to electrostatic interactions with negatively charged cell membranes but may increase plasma protein adsorption and clearance by the immune system [87]. Modifications such as polyethylene glycol grafting effectively shield surface charges and enhance circulation by reducing opsonization [88].

Particle size and polydispersity index (PDI) also exert a major impact on biodistribution, clearance, and tumor penetration [89,90]. Typically, liposomes designed for systemic delivery in cancer therapy have mean diameters ranging from 80 to 200 nm, a range that facilitates passive accumulation in tumor tissue via the EPR effect while avoiding rapid renal clearance [91,92]. Smaller liposomes (<100 nm) may exhibit deeper tumor penetration, whereas larger ones may provide prolonged circulation but reduced diffusivity. The PDI value should ideally be below 0.2 to indicate a homogeneous size distribution, which is important for reproducible biological behavior [93,94].

Although the EPR effect has been widely demonstrated in preclinical tumour models, quantitative analyses in humans indicate that, on average, less than 1% of the injected nanoparticle dose reaches solid tumours, with substantial interpatient and intratumour heterogeneity [95,96]. More recently, intravital imaging and mechanistic studies have shown that active endothelial transcytosis, rather than passive extravasation through inter-endothelial gaps, is a dominant route of nanoparticle entry into solid tumours [97].

The modulation of the lipid bilayer allows the design of liposomes with tailored release profiles, membrane rigidity, and responsiveness to environmental stimuli [98,99]. For melanoma therapy, thermosensitive and pH-sensitive liposomes are of particular interest, as the tumor microenvironment often exhibits acidic pH and increased local temperature due to metabolic hyperactivity [100,101,102]. By incorporating lipids with phase transition temperatures near physiological levels (e.g., DPPC) or pH-sensitive components such as cholesteryl hemisuccinate, researchers can engineer formulations that release their payload selectively at the tumor site [103,104].

Collectively, these physicochemical characteristics define the therapeutic potential of liposomal nanocarriers and enable their rational design for targeted melanoma therapy [105,106]. A deep understanding of these parameters is essential for the development of liposomal platforms capable of navigating the complex biological barriers of cutaneous tumors and delivering therapeutic agents effectively to malignant cells [107,108,109].

## 5. Targeting Strategies: Passive vs. Active Targeting Mechanisms in Cutaneous Tumors

Liposomes designed for melanoma therapy can reach tumor sites via either passive or active targeting mechanisms, each governed by distinct physiological and molecular principles [110,111,112,113]. Passive targeting relies on the EPR effect, which allows nanoscale particles (typically between 50 and 200 nm) to extravasate through the leaky vasculature characteristic of solid tumors and accumulate within the tumor microenvironment due to impaired lymphatic drainage [107,113,114]. Multiple studies have demonstrated that PEGylated liposomes with sizes below 200 nm exhibit significantly higher tumor accumulation and prolonged circulation time, optimizing their passive delivery capacity [109,114]. PEGylation contributes to this effect by forming a hydrophilic steric barrier on the liposome surface, thereby reducing opsonization and uptake by the mononuclear phagocyte system (MPS), which extends plasma half-life and increases the probability of tumor extravasation [115,116].

However, the EPR effect is known to vary greatly across tumor types and individual patients, leading to inconsistent drug delivery outcomes [91,94,112]. To overcome these limitations, active targeting strategies have been increasingly investigated [112]. These involve the surface functionalization of liposomes with ligands such as monoclonal antibodies [117,118,119], antibody fragments [95,96], peptides [97,120,121,122], or small molecules that specifically bind to overexpressed receptors on tumor or stromal cells [19,123,124]. This ligand–receptor interaction facilitates receptor-mediated endocytosis and enhances cellular uptake, increasing therapeutic precision and intracellular drug accumulation [125].

Among the most studied ligands are RGD peptides, which bind to α_vβ_3 and α_vβ_5 integrins receptors overexpressed in melanoma cells and angiogenic endothelial cells [122,126]. Recent studies have shown that RGD-functionalized liposomes significantly improve cellular internalization and tumor accumulation, offering a promising platform for theranostic applications that integrate targeted therapy and imaging [127,128].

Other relevant ligands include full antibodies or antibody fragments (Fab), used to target clinically relevant receptors such as EGFR, HER2, and transferrin receptors. Fab fragments, in particular, present advantages in terms of reduced immunogenicity and improved tissue penetration compared to full antibodies [129,130,131].

Additionally, low molecular weight ligands like folate and transferrin have been employed to target receptors frequently overexpressed in melanoma and other solid tumors [132,133]. Folate receptors, for example, are highly expressed in certain melanoma subtypes, while transferrin receptors exploit the increased iron demand of proliferating cancer cells [133].

Overall, actively targeted liposomes represent a significant advancement in overcoming the limitations of passive targeting by enhancing tumor specificity and intracellular drug delivery. Current trends favor hierarchical strategies that combine passive EPR-based accumulation with active ligand-mediated internalization, as well as responsiveness to endogenous or exogenous stimuli. Such multifunctional systems hold the potential to maximize therapeutic efficacy while minimizing off-target effects and systemic toxicity [91,92,94,112].

## 6. Encapsulated Agents: Chemotherapeutics, Immunomodulators, Nucleic Acids

The structural and functional versatility of liposomes allows for the incorporation of a wide range of therapeutic agents, making them highly adaptable platforms for the treatment of complex neoplasms such as melanoma [134]. Among the compounds most frequently encapsulated in liposomal formulations for melanoma are classical cytotoxic drugs, immunomodulators, and therapeutic nucleic acids, either individually or in combination protocols [135,136,137]. Encapsulation enhances the stability, bioavailability, and selectivity of these agents, reducing systemic side effects while increasing the intratumoral concentration of the active substances [135].

Doxorubicin remains one of the most extensively studied chemotherapeutic agents for melanoma treatment. Liposomes containing doxorubicin, such as Doxil^®^, have shown efficacy in preclinical melanoma models by promoting controlled drug release within the tumor microenvironment. This targeted delivery enhances cytotoxic effects while reducing the cardiotoxicity commonly associated with free doxorubicin [138,139,140]. Another commonly studied agent is paclitaxel, a microtubule-stabilizing compound whose toxicity and low solubility are mitigated through liposomal encapsulation [141,142,143]. In murine melanoma models, liposomal paclitaxel has demonstrated significant tumor cell apoptosis and improved tolerability [142]. Other promising drugs like cisplatin, temozolomide, and vindesine have also been delivered via conventional or PEGylated liposomal systems to optimize their pharmacokinetic profiles [134].

In immunotherapy, liposomes serve as effective carriers for immunomodulators, functioning either as vaccine adjuvants or as delivery vehicles for monoclonal antibodies. Liposomal formulations containing interleukin-2 (IL-2) or interferon-α have shown potential to activate T cells and enhancing tumor-specific immune responses. Additionally, liposomes functionalized with antibodies against PD-1, PD-L1, or CTLA-4, known as immunoliposomes, have been developed to actively target tumor and immune cells [144,145,146,147]. These approaches not only exert direct cytotoxicity but also aim to overcome the immune suppression frequently observed in the melanoma microenvironment [144].

In the context of gene and RNA-based therapies, liposomes have proven to be efficient platforms for the delivery of nucleic acids, such as siRNA, microRNA, and mRNA [148]. Encapsulation of siRNA targeting oncogenic mutations such as BRAF or NRAS has been extensively explored, demonstrating reduced proliferation, induction of apoptosis, and decreased metastatic potential [148,149]. mRNA-based vaccines encapsulated in liposomes are currently developed to elicit adaptive immune responses against tumor-specific antigens, including MART-1, gp100, and tyrosinase [148]. These formulations build upon technologies developed for cationic or ionizable liposomes used in approved mRNA vaccines, adapted to oncological contexts. In murine melanoma models, such systems have demonstrated not only robust immunogenicity but also substantial tumor burden reduction [150,151].

Moreover, co-encapsulation strategies have been investigated to combine multiple therapeutic mechanisms within a single liposomal formulation. For instance, the simultaneous delivery of doxorubicin and siRNA in multifunctional liposomes enables direct cytotoxic activity while modulating specific signaling pathways, thus enhancing antitumor efficacy and overcoming resistance mechanisms [138,149].

Overall, the use of liposomes as vectors for chemotherapeutics, immunomodulators, and nucleic acids currently constitutes one of the most promising approaches in nanotechnology applied to melanoma treatment [17,69,78,80,116].

## 7. Stimuli-Responsive and Multifunctional Liposomes

Stimuli-responsive liposomes represent a particularly innovative approach in oncological nanotechnology as they address critical challenges in melanoma treatment such as premature drug leakage, limited tumor penetration, and the need for precise therapeutic targeting within heterogeneous tumor environments [110,152,153]. These systems are designed to respond in a controlled manner to specific physiological changes within the tumor microenvironment or to externally applied triggers, enabling controlled and site-specific drug delivery [152,153].

One commonly exploited internal trigger is the mildly acidic pH characteristic of the tumor milieu (typically between 6.5 and 6.9), contrasted with the neutral pH of healthy tissues (~7.4). This difference has been utilized in the development of pH-sensitive liposomes through the incorporation of acid-labile lipids or polymers such as hydrazone, which break specifically in acidic environments, promoting the release of encapsulated content within tumor regions or in endosomal compartments following cellular internalization [154,155]. Another key endogenous stimulus is the elevated intracellular concentration of glutathione (GSH) in tumor cells, which can be up to 1000 times higher than plasma levels. GSH-sensitive liposomes are formulated with disulfide bonds or redox-responsive materials that undergo reductive cleavage, enabling preferential intracellular drug release within tumor cells [156,157].

Thermo-responsive liposomes have also been extensively investigated, particularly in combination with localized hyperthermia strategies. The inclusion of lipids with defined thermal phase transition points, such as dipalmitoylphosphatidylcholine (DPPC), allows the lipid bilayer to become more fluid and permeable at temperatures around 41–42 °C, thereby enabling rapid and localized drug release at heat-treated tumor sites. This strategy has been explored in murine models of melanoma as well as in clinical trials using liposomal formulations containing doxorubicin [158,159].

In addition to internal triggers, photo-responsive liposomes are gaining interest. These systems incorporate photosensitive molecules that destabilize under specific light exposure (UV, visible, or near-infrared NIR), triggering bilayer disruption and drug release [160,161,162]. This approach allows for noninvasive, externally controlled activation of the therapeutic system, particularly suitable for superficial cutaneous lesions such as primary melanoma. Recent studies have also developed enzyme-responsive liposomes, which include cleavable substrates targeted by enzymes overexpressed in the tumor microenvironment, such as matrix metalloproteinases (MMPs) or phospholipases, thereby increasing the spatial specificity of drug release [163,164].

In parallel with responsive technologies, multifunctional liposomes have emerged that integrate multiple properties within a single platform. These include surface ligand functionalization, stimulus-responsiveness, and the ability to co-encapsulate multiple synergistic therapeutic agents [162,163]. These advanced formulations aim to overcome the limitations of monofunctional systems by enabling simultaneous action on multiple targets, combining, for example, cytotoxic drugs with immunomodulators or siRNA. The incorporation of hybrid nanomaterials such as metal oxides, conductive polymers, or magnetic elements has further expanded the functional response spectrum, supporting combined thermo-/photo-/magneto-responsive strategies [164].

Although still largely in the preclinical stage, stimuli-responsive and multifunctional liposomes represent a promising frontier in melanoma therapy, particularly due to their ability to adapt to the dynamic tumor microenvironment and phenotypic diversity of malignant cells [165]. Continuous advances of these technologies, combined with the use of advanced characterization techniques such as atomic force microscopy, Raman spectroscopy, and super-resolution fluorescence imaging, has contributed to better control of their in vivo behavior and to progress toward clinical translation [166].

## 8. Preclinical Efficacy and Safety in Melanoma Models (Liposomes)

The main characteristics of the preclinical studies included in this review are summarized in Table 1, which compiles melanoma models, liposomal platforms, targeting strategies, dosing approaches, efficacy outcomes, and available safety indicators. This table serves as a descriptive foundation for the preclinical findings discussed in the following paragraphs.

**Figure 4:**
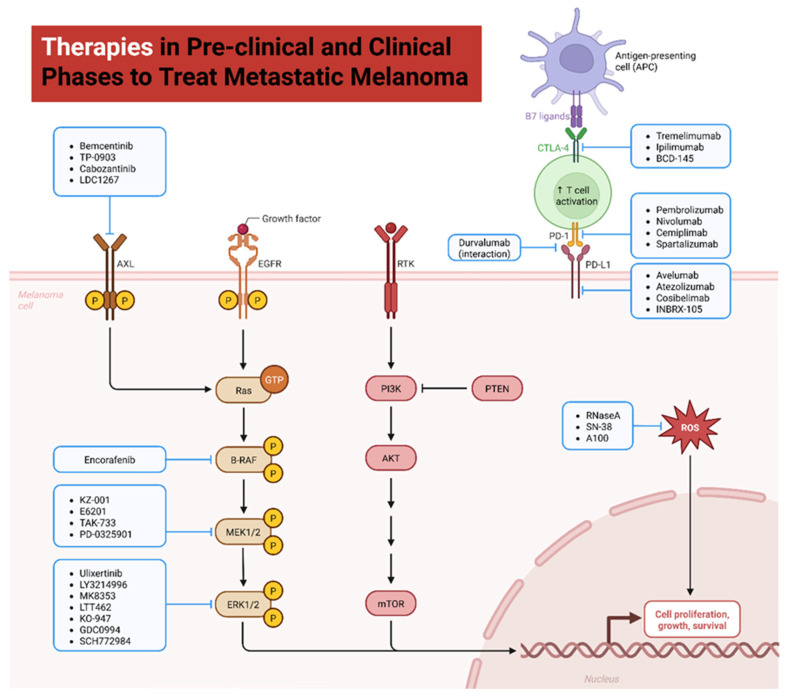
Therapies in pre-clinical and clinical phases to treat metastatic melanoma. Schematic representation of targeted therapies and immunotherapies under investigation, including inhibitors of the MAPK and PI3K/AKT/mTOR pathways as well as immune checkpoint modulators (anti-PD-1, anti-PD-L1, and anti-CTLA-4 antibodies). This integrative map highlights the current therapeutic landscape and supports the rationale for liposomal platforms as innovative nanocarriers to enhance efficacy and reduce systemic toxicity.

The preclinical efficacy and safety of liposomal formulations for melanoma treatment have been extensively demonstrated in both in vitro and in vivo models, with notable outcomes in tumor reduction, cytotoxic selectivity, and immune modulation [167]. In murine models using B16-F10 melanoma cells, the administration of liposomal doxorubicin led to up to a 78% tumor volume reduction after 14 days of treatment, compared to 42% achieved with the free drug. Furthermore, the median survival of treated animals increased from 19 to 32 days [168]. Similarly, PEGylated liposomal paclitaxel demonstrated superior efficacy in A375 melanoma models, resulting in a 2.3-fold increase in tumor cell apoptosis compared to conventional paclitaxel (Figure 4) [169].

The predominant mechanism of action reported is receptor-mediated endocytosis, particularly after liposome surface functionalization with targeting ligands such as RGD peptides (which bind to αvβ3 integrins) or anti-PD-1 antibodies. In a study, anti-PD-1 immunoliposomes loaded with doxorubicin induced complete tumor regression in 40% of the treated mice, whereas no complete regressions were observed in control groups [170]. Additionally, pH or redox-responsive formulations sensitive to the acidic (~6.5) or glutathione-rich microenvironment of melanoma achieved 3 to 5-fold higher intratumoral drug concentrations compared to non-responsive liposomes [171].

In the context of immunotherapy, liposomes encapsulating mRNA encoding tumor-associated antigens (such as TRP-2 or gp100), combined with lipid-based adjuvants like MPLA, promoted up to a 6-fold increase in CD8^+^ T cell proliferation and significantly elevated serum IFN-γ levels compared to non-encapsulated controls [172]. These findings support the role of liposomes not only as drug carriers but also as immunogenic platforms capable of eliciting robust and durable antitumor immune responses [173].

Regarding safety, studies consistently report a significant reduction in systemic toxicity compared to free drug administration. In one trial, using liposomal temozolomide, treated mice exhibited physiological levels of ALT and creatinine, with no relevant histopathological alterations in the liver, kidneys, or spleen after 21 days of treatment [174]. These outcomes reflect the high biocompatibility of liposomal systems, especially when combined with PEGylation and controlled particle sizes typically ranging from 80 to 150 nm [175].

Collectively, current preclinical data underscore that optimized liposomes offer substantial advantages over conventional treatments in both antitumor efficacy and safety profile [176]. The convergence of active targeting, microenvironment-responsive release, and immunomodulatory capabilities positions liposomal nanoplatforms as a strategic component in the development of integrated therapies for melanoma [177]. However, translating these results into clinical practice requires further validation through 3D tumor models and early-phase clinical trials, given the biological complexity and interpatient heterogeneity of human melanomas [178,179].

**Table 1:** Overview of the preclinical evidence on liposomal platforms for melanoma. The table presents representative primary in vitro and in vivo studies included for structured data extraction.

Ref.	Melanoma Model	Liposome/Platform (Composition, Size, PDI)	Targeting Ligand/Strategy	Main Therapeutic Outcomes	Toxicity/Safety Markers	Key Quantitative Findings (As Reported in This Review)
[139]	Melanoma preclinical models (in vitro and in vivo; specific line not specified)	Liposomal doxorubicin (e.g., Doxil^®^); detailed lipid composition, size and PDI not specified in this review	Passive targeting via EPR; no specific ligand mentioned	Improved melanoma control with controlled drug release in the tumor microenvironment; reduced cardiotoxicity compared with free doxorubicin	Reduced cardiotoxicity compared with free doxorubicin; no additional laboratory markers detailed	Qualitatively described as effective in melanoma models, with better safety than free drug; no precise numerical values provided in this review
[143]	Murine melanoma models (A375 and other lines; exact panel not fully detailed)	PEGylated liposomal paclitaxel; specific size and PDI not detailed in this review	Passive targeting; PEGylation to prolong circulation	Significant tumor cell apoptosis and improved tolerability relative to conventional paclitaxel	Improved tolerability vs. conventional formulation; no specific organ toxicity markers described	Reported as inducing “significant” apoptosis with better tolerability; no exact percentages provided in this review
[145]	Melanoma models in immunotherapy context (murine; specific line not detailed)	Liposomal formulations containing immunomodulators such as IL-2 or IFN-α; physicochemical parameters not specified	Functional targeting of immune system (T-cell activation); no tumor-specific ligand described	Activation of T cells and enhancement of tumor-specific immune responses in melanoma	No major systemic toxicity reported in the synthesis; detailed markers not specified	Qualitatively associated with enhanced antitumor immunity; no quantitative tumor volume or survival data presented in this review
[147]	Melanoma models exploring checkpoint inhibition (murine)	Immunoliposomes functionalized with antibodies against PD-1, PD-L1 or CTLA-4; size and PDI not specified	Active targeting via surface-bound checkpoint antibodies	Enhanced tumor and immune-cell targeting, aiming to overcome immune suppression in the melanoma microenvironment	Safety profile not quantified; described as a strategy to reduce off-target toxicity	Reported as promising for combining direct cytotoxicity and immune modulation; no numerical tumor or survival data given in this review
[149]	Melanoma models harboring oncogenic BRAF^V600E^ or NRAS mutations (murine/in vitro)	Liposomal delivery systems for siRNA (and related nucleic acids); detailed composition, size and PDI not specified	Functional genetic targeting (siRNA against BRAF^V600E^ or NRAS); no additional ligand reported	Reduced proliferation, induction of apoptosis and decreased metastatic potential in melanoma models	Toxicity profile not detailed; focus is on antitumor effects	Qualitatively associated with reduced tumor aggressiveness; no explicit percentages or survival times provided in this review
[151]	Murine melanoma models (e.g., antigens MART-1, gp100, tyrosinase)	Liposomal or ionizable lipid-based mRNA vaccines; typical particle sizes ~80–150 nm cited for these platforms; PDI not specified per study	Functional targeting of antigen-presenting cells via lipid nanoparticles; no tumor-specific ligand described	Robust adaptive immune responses against melanoma-associated antigens and substantial tumor burden reduction	No major systemic toxicity reported; emphasis on immunogenicity and antitumor effect	Described as producing strong immunogenicity and marked tumor burden reduction; numerical values not detailed in this review
[159]	Murine melanoma models; combined with local hyperthermia	Thermo-responsive liposomes containing doxorubicin; lipids with defined phase-transition temperatures (e.g., DPPC); size/PDI not specified	Thermo-responsive release at 41–42 °C; no additional ligand mentioned	Rapid, localized release of doxorubicin at heated tumor sites, enhancing antitumor activity in melanoma	Safety described as acceptable in preclinical and early clinical investigations; no detailed laboratory data given	Reported as improving local tumor control in melanoma with controlled release under hyperthermia; quantitative tumor/survival data not specified in this review
[160]	Clinical and preclinical contexts including melanoma (hyperthermia-assisted therapy)	Thermo-sensitive liposomal doxorubicin in clinical trial settings; physicochemical details not specified in the review	Thermo-triggered release plus passive targeting; no ligand	Improved local drug release at tumor sites; integration with hyperthermia strategies for solid tumors, including melanoma	Clinical tolerability considered acceptable in early trials; specifics not enumerated	Supports translational potential of thermo-sensitive liposomes; no melanoma-specific clinical response rates provided in this review
[169]	Murine melanoma, B16-F10 cells implanted in mice	Liposomal doxorubicin; detailed lipid composition, size and PDI not described in this review	Passive targeting (no specific ligand mentioned)	Marked tumor growth inhibition and survival benefit compared with free doxorubicin	Systemic toxicity not deeply characterized; focus on survival and tumor volume	Tumor volume reduction up to 78% with liposomal doxorubicin vs. 42% with free drug; median survival increased from 19 to 32 days
[170]	Murine melanoma model A375	PEGylated liposomal paclitaxel; detailed composition, size and PDI not provided in the review	Passive targeting with PEGylation to prolong circulation; no specific ligand	Superior efficacy compared to conventional paclitaxel, with enhanced apoptosis in melanoma cells	Safety profile suggested as improved; no detailed toxicity markers described	Approximately 2.3-fold increase in tumor cell apoptosis compared with conventional paclitaxel
[171]	Murine melanoma model (line not specified in the review; anti-PD-1-based strategy)	Anti-PD-1 immunoliposomes loaded with doxorubicin; physicochemical properties not detailed in this review	Active targeting via surface-bound anti-PD-1 antibodies	Robust antitumor response with complete tumor regression in a subset of treated mice	Toxicity not detailed in this review; focus is on efficacy	40% of treated mice achieved complete tumor regression; no complete regressions in control groups
[172]	Preclinical melanoma models with acidic and glutathione-rich microenvironment	pH- and redox-responsive liposomal formulations; size and PDI not specified	Stimuli-responsive design (acidic pH and elevated GSH); no receptor–ligand targeting specified	Enhanced intratumoral drug delivery relative to non-responsive liposomes	No specific systemic toxicity markers reported; focus on distribution	Achieved 3- to 5-fold higher intratumoral drug concentrations than non-responsive liposomes
[173]	Murine melanoma models using tumor-associated antigens such as TRP-2 or gp100	Liposomes encapsulating mRNA encoding tumor-associated antigens + lipid adjuvant MPLA; composition details not fully specified	Functional immunotargeting via mRNA vaccine platform; no tumor-cell receptor ligand described	Potent activation of antitumor immune responses (CD8^+^ T cells, cytokines)	Systemic toxicity not detailed; focus on immune parameters	Up to 6-fold increase in CD8^+^ T-cell proliferation and significantly elevated serum IFN-γ vs. non-encapsulated controls
[175]	Murine model treated with liposomal temozolomide; tumor type includes melanoma within preclinical context	Liposomal temozolomide; typical liposomal sizes 80–150 nm mentioned for systems in this range; PDI not specified for this specific study	Passive targeting (EPR); no active ligand described	Maintained antitumor effect with reduced systemic toxicity compared to free temozolomide	ALT and creatinine remained within physiological levels; no relevant histopathological alterations in liver, kidney or spleen after 21 days	Demonstrated preservation of organ function and histology while sustaining therapeutic effect, supporting improved safety profile vs. free drug

## 9. Clinical Evidence of Liposomal Formulations in Melanoma

Although most data on liposomal systems in melanoma are preclinical, a few clinical studies illustrate both the potential and the current limits of translation (Table 2). One of the earliest experiences comes from Adler et al., who tested an allogeneic human liposomal melanoma vaccine in 24 patients with metastatic disease, in four parallel arms combining or not combining the vaccine with regional or systemic IL-2. In the arm that received vaccine plus low-dose regional IL-2, three complete and three partial responses were observed (6/10 responders), whereas no objective responses occurred with vaccine alone or with vaccine plus systemic IL-2, suggesting that local cytokine support and adequate immune priming are critical for clinical activity [180].

Cytotoxic liposomal chemotherapy has also been explored. Two phase II trials evaluated pegylated liposomal doxorubicin (PLD, Caelyx^®^) in patients with metastatic melanoma who had failed prior dacarbazine-based chemotherapy. In the smaller study by Vorobiof et al. (14 patients; 50 mg/m^2^ every 28 days), treatment was well tolerated but no objective responses were documented, leading the authors to consider PLD monotherapy insufficiently active in this setting [181]. In a larger second-line study with 30 patients, Fink et al. used a similar schedule and reported one complete response, one partial response and five cases of stable disease lasting more than 90 days; median overall survival after PLD initiation was 214 days, again with a favourable toxicity profile but limited antitumor efficacy [182].

More recent efforts have focused on immunogenic liposomal platforms. Lipovaxin-MM, a dendritic-cell–targeted multi-component liposomal vaccine, was evaluated in a phase I 3 + 3 dose-escalation trial including 12 patients with metastatic cutaneous melanoma. The vaccine was well tolerated, with mostly grade 1–2 adverse events; one partial response and two cases of stable disease were documented, but robust vaccine-specific T-cell or antibody responses were not consistently detected, indicating that further optimisation of antigen loading and immune stimulation is needed [183].

The most advanced clinical programme is BNT111 (FixVac), an intravenously administered RNA-lipoplex vaccine encoding four melanoma-associated antigens. In the phase I Lipo-MERIT trial (NCT02410733), 89 patients with advanced melanoma, many previously exposed to checkpoint inhibitors, received BNT111 alone or combined with anti-PD-1 therapy. The study showed a favourable safety profile and strong induction of antigen-specific CD4^+^ and CD8^+^ T-cell responses, with durable objective responses in a subset of checkpoint-inhibitor–experienced patients [184]. On the basis of these results, a randomized phase II trial (NCT04526899; BNT111-01) was launched in approximately 120 patients with anti-PD-1-refractory or relapsed unresectable stage III/IV melanoma. Interim company reports indicate that the primary endpoint of overall response rate was met, with an improvement versus historical controls and an acceptable safety profile, although full peer-reviewed data are still pending [185].

Taken together, these studies show that liposomal systems have reached the clinic in melanoma, mainly as vaccine and PLD formulations. However, response rates remain modest in chemotherapy-based regimens, and even the more immunogenic RNA-lipoplex platforms are still in early-phase development. The available trials support the biological plausibility and safety of liposomal strategies but do not yet justify strong claims of clinical readiness; larger, controlled phase II–III studies will be necessary to define their real contribution to melanoma therapy (Table 2).

## 10. Discussion

The application of nanotechnology in the treatment of cutaneous melanoma, particularly through liposomal formulations, represents one of the most promising frontiers in contemporary oncology. In light of the limitations posed by conventional therapies such as systemic toxicity, tumor resistance, and low selectivity, liposomes emerge as versatile, biocompatible, and potentially customizable platforms for targeted drug delivery.

This review demonstrates that liposomes offer substantial advantages in terms of pharmacokinetic stability, molecular protection, the ability to encapsulate both hydrophilic and lipophilic compounds, and, most importantly, the capacity for both passive targeting via the enhanced EPR effect and active targeting through surface functionalization with specific ligands [1,7]. The incorporation of stimuli-responsive strategies whether to internal factors (such as pH and glutathione) or external triggers (such as light and temperature) further enhances the precision and efficacy of these systems, allowing for localized and controlled drug release [154,155,159].

Therapeutically, liposomes have been explored as carriers for classical cytotoxic drugs, immunomodulators, and nucleic acids, with increasing interest in multifunctional formulations that combine several therapeutic mechanisms, such as co-encapsulation of siRNA and chemotherapeutic agents [156,157]. Preclinical studies have shown significant tumor reduction, increased survival, and lower systemic toxicity in murine models of melanoma [169,175]. Moreover, their ability to elicit robust immune responses, as seen in liposomes containing tumor-associated mRNA and lipid-based adjuvants, reinforces their potential as immunogenic platforms [173,174].

Nevertheless, major challenges remain in translating these technologies to clinical practice, including issues of dermal penetration, intertumoral heterogeneity, regulatory complexity, and industrial scalability [25,26]. Addressing these challenges will require integrated efforts encompassing advanced formulation engineering, deeper understanding of melanoma biology, pharmacogenomic approaches, and improved three-dimensional tumor models that more accurately reflect human melanoma pathophysiology [165].

Future developments in liposomal nanotechnology for melanoma will likely depend on advances in microenvironment-responsive designs, improved penetration strategies, and more predictive human-relevant models such as 3D melanoma constructs and organoids. Integrating pharmacogenomic profiling, scalable manufacturing approaches, and rational ligand selection may accelerate translation. Although current clinical evidence remains limited, the growing precision of liposomal engineering suggests meaningful opportunities for safer, more selective and personalized melanoma therapies.

In summary, although still largely experimental, liposomal platforms constitute a rapidly advancing area in cutaneous melanoma therapy. Continued progress in nanocarrier design, combined with a deeper understanding of the tumor microenvironment, position liposomes as central components in the development of more selective, effective, and personalized therapies for one of the most aggressive types of skin cancer.

## 11. Methods

This systematic review was conducted in accordance with the PRISMA 2020 guidelines following predefined procedures for search, screening, eligibility assessment, and qualitative synthesis. The objective was to identify, organize and critically interpret current scientific evidence on the application of liposomal nanotechnology in targeted therapy for cutaneous melanoma.

### 11.1. Search Strategy

A comprehensive search was conducted in four major databases—PubMed, Scopus, Web of Science and EMBASE—covering publications from January 2010 to February 2025. Search strategies combined Boolean operators with controlled vocabulary terms (MeSH and Emtree), adapted to the indexing characteristics of each database. The initial search retrieved 1284 records (PubMed n = 412; Scopus n = 365; Web of Science n = 281; EMBASE n = 226). After removal of 326 duplicates, 958 records remained for title and abstract screening.

Database: PubMed
Time frame: January 2010 to February 2025
Language restriction: English
Last search date: 15 February 2025
(“melanoma”[MeSH Terms] OR melanoma[Title/Abstract])
AND
(“liposomes”[MeSH Terms] OR liposomal[Title/Abstract])
AND
(nanotechnology OR nanomedicine OR “drug delivery”)
No additional study design filters were applied at the search stage. Equivalent strategies were adapted for Scopus, Web of Science, and EMBASE according to their specific syntax and indexing requirements. The final search was performed on 25 August 2025.

### 11.2. Eligibility Criteria

Articles were selected based on the following inclusion criteria:

(a) availability of full text;
(b) publication in English;
(c) original research articles or reviews addressing liposomal nanocarriers applied to melanoma;
(d) investigation of liposomal formulation, delivery mechanisms, preclinical or clinical outcomes, or immunoliposome-based strategies.

Exclusion criteria included:

(a) studies focused exclusively on non-melanoma skin cancers;
(b) reports involving nanoparticle systems other than liposomes, except when they served as comparative arms;
(c) editorials, commentaries, conference abstracts, letters, and duplicate entries;
(d) studies in which melanoma was only marginally mentioned or not treated as a central analytical focus.

### 11.3. Literature Selection and Data Extraction

Two independent reviewers screened all titles and abstracts. Articles that met inclusion criteria or could not be confidently excluded proceeded to full-text evaluation. Disagreements were resolved through discussion and consensus.

The PRISMA selection process unfolded as follows:

- Records identified: 1284
- Duplicates removed: 326
- Records screened: 958
- Records excluded at screening: 742
- Full-text articles assessed: 221
- Full-text exclusions: 62 due to non-liposomal nanoparticle systems;

41 for insufficient methodological detail;

28 for addressing melanoma only peripherally;

15 due to language restrictions.

A total of 159 studies met all eligibility criteria and were included in the qualitative synthesis. These 159 studies constitute the analytic foundation of the systematic review.

**Figure 5:**
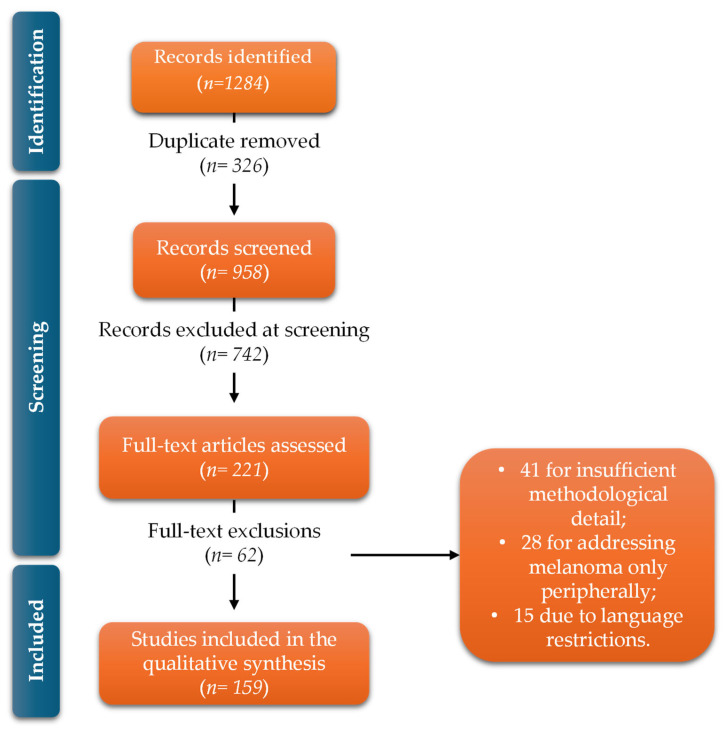
PRISMA 2020 flow diagram summarizing the identification, screening, eligibility assessment and inclusion of studies, resulting in 159 articles incorporated into the qualitative synthesis.

In total, 185 articles were cited in the manuscript:

- 26 articles were used solely to contextualize the Introduction, typically covering epidemiology, molecular mechanisms, tumor classification and the current therapeutic landscape;
- 159 articles correspond to the studies included in the systematic review;

A PRISMA 2020 flow diagram is presented in Figure 5.

### 11.4. Data Extraction

For each of the 159 included studies, data extraction followed a structured approach. Extracted variables included:

- lipid composition and physicochemical characteristics of the liposomal formulation;
- therapeutic cargo (chemotherapeutics, immunomodulators, nucleic acids);
- delivery approach (passive vs. active targeting);
- biological model (in vitro assays, animal models, or clinical studies);
- primary therapeutic outcomes (cytotoxicity, tumor regression, immunomodulation);
- Clinical trials and translational challenges safety assessments (cell viability, organ toxicity, histopathological analysis);
- translational implications and scalability.

Data extraction was performed independently by two reviewers, with cross-checking to ensure consistency.

### 11.5. Risk of Bias Assessment

Given the predominantly preclinical nature of the evidence, risk of bias (RoB) was assessed using design-appropriate tools. In vitro studies were evaluated using ToxRTool, focusing on methodological reliability and reporting quality. Animal studies were assessed using SYRCLE’s RoB tool, addressing selection, performance, detection, and reporting biases. Studies with mixed mechanistic endpoints were evaluated using adapted OHAT criteria.

Early-phase clinical studies were not subjected to formal RoB tools due to inherent design limitations and were interpreted descriptively. Overall, the most common sources of bias were incomplete reporting of randomization, allocation concealment, and blinding, leading to a predominance of unclear risk of bias across preclinical studies (Table 3).

Risk of bias assessment was applied only to primary experimental and early-phase clinical studies. A total of 45 included articles consisted of review, conceptual, or translational papers and were therefore not subjected to formal risk of bias evaluation, in accordance with PRISMA recommendations.

### 11.6. Certainty of Evidence

Given the heterogeneity in formulations, biological models and outcome definitions, meta-analysis was not feasible. Certainty of evidence was synthesized qualitatively following principles adapted from GRADE for preclinical research.

- Evidence for liposomal doxorubicin and liposomal paclitaxel demonstrated moderate certainty, supported by consistent antitumor effects across animal models;
- Studies involving nucleic acid-based liposomes and immunoliposomes were assigned low certainty, largely due to variability in targeting strategies and immune endpoints;
- Stimuli-responsive and multifunctional liposomes were classified as very low certainty, as these technologies remain in early experimental stages and are represented by a small number of heterogeneous studies.

### 11.7. Thematic Synthesis of Included Studies

Following content analysis, the 159 included studies were categorized into six major thematic domains:

1. liposomal formulations and physicochemical characteristics relevant to melanoma therapy;
2. passive and active targeting strategies applied to cutaneous tumors;
3. therapeutic payloads, including chemotherapeutics, immunomodulators and nucleic acids;
4. stimuli-responsive and multifunctional liposomes;
5. preclinical efficacy and safety in melanoma models;
6. clinical and translational challenges, including scalability and regulatory considerations.

## Figures and Tables

**Table 2:** Clinical trials investigating liposomal formulations in melanoma.

Trial ID	Phase	Liposomal Formulation	Study Design/Population	Regimen	Primary Endpoint	Key Findings
[181]	Pilot (early phase)	Allogeneic human melanoma-membrane liposomes ± regional or systemic IL-2	24 patients with metastatic melanoma, four concurrent arms (vaccine alone; vaccine + systemic IL-2; vaccine + regional low-dose IL-2; regional IL-2 alone)	Repeated vaccine injections; IL-2 given either systemically or regionally at low dose depending on arm	Clinical and immunological response	In the vaccine + regional IL-2 arm, 3 CR and 3 PR among 10 patients; 3 PR in IL-2–only arm; no responses with vaccine alone or vaccine + systemic IL-2. Responders developed delayed-type hypersensitivity and enhanced in vitro cytotoxicity against melanoma cells.
[182]	Phase II	Pegylated liposomal doxorubicin (Caelyx^®^)	14 patients with metastatic melanoma refractory to dacarbazine-based chemotherapy; single-arm study	50 mg/m^2^ IV every 28 days	Objective response rate; safety	No objective responses observed; treatment generally well tolerated. Authors concluded that PLD monotherapy showed insufficient activity in this heavily pretreated setting.
[183]	Phase II	Pegylated liposomal doxorubicin (Caelyx^®^)	30 patients with progressing disseminated melanoma after prior chemotherapy; second-line, single-arm	50 mg/m^2^ IV on days 1, 22, 43, 64, then 40 mg/m^2^ every 4 weeks	Response rate, survival, time-to-progression, toxicity	Treatment well tolerated; erythrodysesthesia grade 3 in 6%. One CR, one PR and five SD > 90 days. Median survival 214 days (95% CI 151–304), indicating limited efficacy but possible benefit in a subset.
[184]	Phase I	Dendritic-cell–targeted multi-component liposomal vaccine (Lipovaxin-MM)	12 patients with metastatic cutaneous melanoma; 3 + 3 dose-escalation design in three cohorts	0.1 or 1 mL every 4 weeks (cohorts A/B) or 3 mL weekly × 4 (cohort C), IV	Safety, dose-limiting toxicity, immunogenicity, preliminary efficacy	No dose-limiting toxicities; most adverse events grade 1–2. One PR and two SD; no consistent vaccine-specific T-cell or antibody responses detected, indicating good tolerability but limited and variable clinical activity.
[185]	Phase I	RNA-lipoplex vaccine (BNT111) encoding four melanoma-associated antigens	Multicentre, dose-escalation trial in 89 patients with advanced melanoma, many pretreated with checkpoint inhibitors; BNT111 alone or with anti-PD-1	IV administration of BNT111 at escalating doses; with or without anti-PD-1 antibody	Safety, tolerability, immunogenicity; exploratory antitumor activity	Favourable safety profile; strong CD4^+^/CD8^+^ T-cell responses against vaccine antigens; durable objective responses observed in a subset of checkpoint-inhibitor–experienced patients, especially when combined with PD-1 blockade.

**Table 3:** Summary of risk of bias across included studies.

Study Type (n)	Risk-of-Bias Tool/Framework	Key Domains Considered	Overall Risk-of-Bias Overview (High-Level)
In vitro studies (n = 46)	ToxRTool	Reporting of experimental procedures, relevance of model/assay, appropriate controls, reproducibility (e.g., replicates), outcome measurement and data presentation	Overall moderate risk of bias, driven mainly by incomplete reporting of critical methodological details (e.g., replicates/independent repeats, blinding where applicable, and clarity of outcome measurement).
Animal studies (in vivo) (n = 38)	SYRCLE’s Risk of Bias tool	Sequence generation (randomization), allocation concealment, blinding (performance/detection), incomplete outcome data, selective reporting, other biases	Overall unclear-to-moderate risk of bias, predominantly due to unclear reporting of randomization, allocation concealment, and blinding procedures.
Combined in vitro/in vivo studies (n = 21)	Adapted OHAT criteria	Internal validity across mixed endpoints (mechanistic + biological outcomes), consistency and transparency of methods, outcome assessment, reporting completeness	Overall moderate risk of bias, reflecting methodological heterogeneity across endpoints and variable reporting completeness across experimental components.
Early-phase clinical studies (Phase I–II) (n = 9)	Descriptive appraisal (design-appropriate RoB considerations)	Selection/eligibility clarity, intervention description, outcome definitions, follow-up completeness, reporting transparency	Formal RoB tools were not applied; these studies were considered inherently imprecise due to exploratory design and small sample sizes, with risk primarily related to limited power and non-comparative designs.

Abbreviations: OHAT, Office of Health Assessment and Translation; RoB, risk of bias.

## Data Availability

No new data were created or analyzed in this study. Data sharing is not applicable to this article.
